# Evolution of Tribotronics: From Fundamental Concepts to Potential Uses

**DOI:** 10.3390/mi15101259

**Published:** 2024-10-15

**Authors:** Yue He, Jia Tian, Fangpei Li, Wenbo Peng, Yongning He

**Affiliations:** 1 School of Microelectronics, Xi’an Jiaotong University, Xi’an 710049, China; 2 The Key Lab of Micro-Nano Electronics and System Integration of Xi’an City, Xi’an 710049, China

**Keywords:** tribotronics, contact electrification (CE), triboelectric nanogenerator (TENG), field-effect transistor (FET), self-powered device

## Abstract

The intelligent sensing network is one of the key components in the construction of the Internet of Things, and the power supply technology of sensor communication nodes needs to be solved urgently. As a new field combining tribo-potential with semiconductor devices, tribotronics, based on the contact electrification (CE) effect, realizes direct interaction between the external environment and semiconductor devices by combining triboelectric nanogenerator (TENG) and field-effect transistor (FET), further expanding the application prospects of micro/nano energy. In this paper, the research progress of tribotronics is systematically reviewed. Firstly, the mechanism of the CE effect and the working principles of TENG are introduced. Secondly, the regulation theory of tribo-potential on carrier transportation in semiconductor devices and the research status of tribotronic transistors are summarized. Subsequently, the applications of tribotronics in logic circuits and memory devices, smart sensors, and artificial synapses in recent years are demonstrated. Finally, the challenges and development prospects of tribotronics in the future are projected.

## 1. Introduction

With the arrival of Industry 4.0, the information society is progressing towards the era of the Internet of Everything. In the construction of the Internet of Things (IoT), sensor nodes are recognized as one of the most critical components [1,2,3]. With the development of IoT technology and integrated circuit technology, the integration level of electronic devices continues to increase. However, power supply technology for sensing communication nodes remains a significant limitation hindering the deployment of distributed intelligent sensing networks, primarily because traditional power supply schemes are unsuitable for the numerous small-sized sensor nodes [4,5,6,7]. In 2012, Prof. Zhong Lin Wang and his team introduced the triboelectric nanogenerator (TENG), which converts stray mechanical energy into electrical energy through the contact electrification (CE) effect and electrostatic induction, thereby presenting a promising solution for self-powered electronic devices [8,9,10]. However, the high internal resistance of the traditional insulator-based TENG (~MΩ/GΩ) is incompatible with most electronic devices. Additionally, TENG produces high-voltage alternating current (AC) that requires conversion before practical application, inhibiting their large-scale integration in smart sensing networks [11,12,13].

Based on the electromechanical conversion characteristics of TENG, in 2014, Zhang introduced the concept of tribotronics, which leverages the triboelectric potential generated by TENG as the gate voltage regulation to control the motion of carriers in semiconductors, thus realizing the control of electronic devices through mechanical motion [14]. By coupling triboelectricity with semiconductors, this approach not only solves the compatibility problem between TENG and electronics but also establishes a direct interaction between the external environment and semiconductor devices, which results in the derivation of numerous tribotronic transistors and related functional devices and provides innovative opportunities for applications such as smart sensing networks. Therefore, this review systematically examines the progress of research related to tribotronics. First, we introduce the origin of tribotronics, including the mechanisms of CE and TENG with different working modes. Second, we review the theory of tribo-potential regulation of carrier transportation in semiconductor devices and the research status of tribotronic transistors, especially the tribotronic field-effect transistors (TFET). Subsequently, we highlight recent applications of tribotronics in logic circuits and memory devices, smart sensors, artificial synapses, and some other novel applications. Finally, we present the future challenges and suggestions for the emerging field of tribotronics.

## 2. Origin of Tribotronics

### 2.1. Contact Electrification (CE) Effect

The first TENG device was reported by Prof. Zhong Lin Wang’s team in 2012 [8]. As a novel approach to energy harvesting, TENG operates on the coupled principle of CE and electrostatic induction, enabling the efficient conversion of widespread and stray mechanical energy into electrical energy at the micro/milliwatt scale [15]. This capability presents a highly promising solution for the sustainable power demands of smart sensor networks. The underlying mechanism of TENG, namely CE (or triboelectrification), is a complex physicochemical process. CE is a widely observed natural process wherein materials—ranging from metals and semiconductors to inorganic substances and polymers—can be electrically charged simply through contact [16]. However, the specific physical processes governing CE have remained contentious over the years, primarily due to the absence of appropriate research tools. Recent advancements in characterization techniques, such as X-ray photoelectron spectroscopy, atomic force microscopy, and TENG devices, have significantly enhanced our understanding of charge transfer at CE interfaces and its associated processes [17,18,19,20].

**Figure 1:**
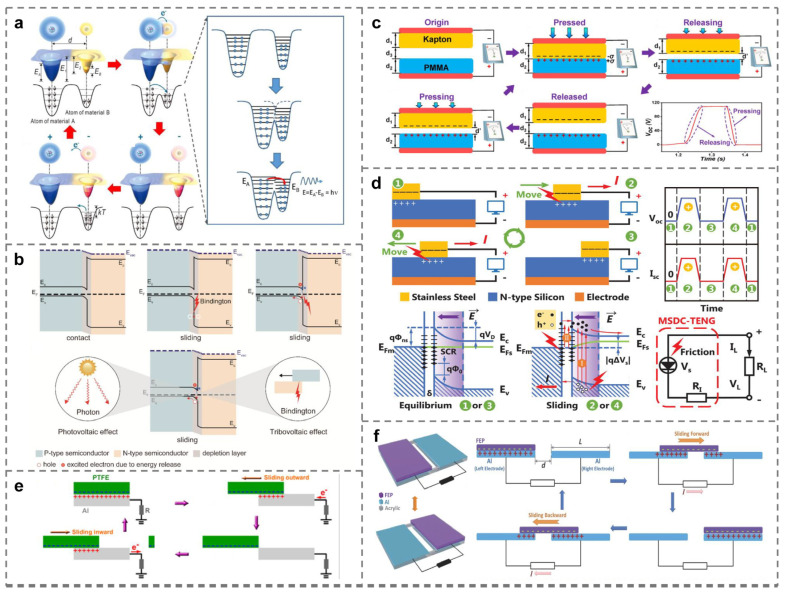
Mechanisms and working modes of TENG. (**a**) The electron-cloud potential-well model proposed for general material cases [21]. (**b**) Energy band diagram of the tribovoltaic effect [32]. (**c**–**f**) Four basic working modes of TENG. (**c**) Contact–separation mode TENG (CS-TENG) [42]. (**d**) Lateral-sliding mode TENG (LS-TENG) [45]. (**e**) Single-electrode mode TENG (SE-TENG) [47]. (**f**) Freestanding triboelectric-layer mode TENG (FT-TENG) [49].

To elucidate the physical processes of CE, Prof. Zhong Lin Wang proposed the electron-cloud potential-well model [21], as shown in Figure 1a: Before the macroscopic contact occurs, two isolated atoms cannot transfer electrons owing to the constraints of their respective potential wells. Upon application of an external force, when the atomic separation reaches an equilibrium distance, their electron clouds overlap, which can be conceptualized as bonding. As the external force further compresses the atomic spacing, the resulting increase in interatomic repulsion coincides with greater electron cloud overlap. This change causes the system to transition from a single potential-well state to an asymmetric double potential-well state. The substantial overlap of the electron clouds results in a reduction in energy barriers between the atoms, thus increasing the likelihood of electron transfer at the atomic scale [22,23,24]. When the two materials are separated, the transferred electrons face challenges crossing the potential barrier to return to their original states without additional energy input (e.g., by increasing the temperature), which would enable the electrons to escape their potential wells and revert to their initial orbitals or dissipate into the surrounding medium. The electron-cloud potential-well model is not only applicable to solid–solid contact interfaces, such as metal–insulator and insulator–insulator but also to liquid–solid and liquid–liquid interfaces [25,26,27,28].

In the context of CE phenomena occurring at semiconductor interfaces, a distinct manifestation is observed compared to conventional CE: a direct current (DC) output consistently emerges from semiconductor-based TENG [29,30,31]. Prof. Zhong Lin Wang named this phenomenon of DC generation at semiconductor interfaces under triboelectric conditions as the tribovoltaic effect [21]. The tribovoltaic effect arises from the coupling of physical processes such as interfacial contact initiation, atomic bonding, and phonon excitation [32]: as shown in Figure 1b, when two semiconductor interfaces come into contact, the contact interface undergoes the breakage of the chemical bond and the formation of a new bond due to the mechanical action, and a large amount of energy will be released in the process (abbreviated as bindington), leading to the excitation of electron–hole pairs and the directional separation and movement of electrons and holes under the action of an electric field, generating direct current [33,34,35]. However, it remains unresolved whether the separation of the electron–hole pairs is predominantly driven by the built-in electric field [36,37,38] or by the interfacial electric field [39,40,41].

### 2.2. Triboelectric Nanogenerator (TENG)

According to different electrode arrangements and motion states, TENG can generally be categorized into four working modes: contact–separation (CS) mode [42,43], lateral-sliding (LS) mode [44,45], single-electrode (SE) mode [46,47], and freestanding triboelectric-layer (FT) mode [48,49]. Each of these four modes operates based on distinct principles. For instance, in the case of CS-TENG, when subjected to a periodic external force in the vertical direction, the two tribo-layers, which are initially separated, periodically come into contact and separate. This process induces charge exchange due to the differential charge-capturing ability of the material surfaces, known as CE. Consequently, the back of the metal electrode generates induced charges through electrostatic induction, resulting in a potential difference between the two electrodes that drive current flow in the external circuit, as illustrated in Figure 1c. In the LS-TENG, energy generated from the sliding motion excites electron–hole pairs at the contact interface, generating a triboelectric potential and driving the external circuit to generate a DC current, depicted in Figure 1d. The operating mechanism of SE-TENG is similar to that of the LS-TENG, but the potential driving the external circuit results from the difference between the potential of the single electrode and that of the ground. Consequently, during cyclic sliding, the potential reverses, resulting in AC output, as shown in Figure 1e. Finally, the FT-TENG is a structure consisting of two SE-TENGs connected by a common tribo-layer. When the tribo-layer slides, different amounts of triboelectric charge are generated on the two electrodes due to the difference in contact area, thus generating a sufficient potential difference to drive the electron transfer in the external circuit, as shown in Figure 1f.

Based on these four modes, a wide variety of structured TENG have been gradually developed to meet diverse practical applications [50,51,52,53]. However, due to the high impedance (~MΩ/GΩ) of TENG itself, which is incompatible with electronic devices [43,54], it cannot be directly used for powering electronic devices. Consequently, additional power management is required to lower the impedance and optimize current integration, which can complicate self-powered device scenarios. Recently, the emergence of tribotronics combining TENGs with transistors has provided innovative strategies for enhancing self-powered sensing networks, thereby bolstering academic rigor in this field.

## 3. Tribotronics and Tribo-Potential Modulated Devices

### 3.1. Fundamentals of Tribotronics

Conventional FET is a voltage-controlled three-terminal device based on the principle that the gate controls the carriers’ motion by creating or eliminating a channel between the source and drain, thereby generating a directional electric field inside the device, so the FET device requires an external circuit to provide the gate voltage to adjust its electrical characteristics, which inevitably brings additional power consumption and poses the challenge of supplying energy for building smart sensing networks. As an extension of the TENG field, the concept of tribotronics was first put forward by Zhang and his team in 2014 by combining the CE effect with the basic theory of semiconductor devices [14]. As mentioned above, TENG can effectively convert mechanical energy into electrical energy. When a capacitive load is connected to the output of TENG, a corresponding holding voltage will be generated across the load, which is also called the tribo-potential [55]. If the tribo-potential is used to generate an electric field in a specific direction and control the transportation state of carriers, the output performance of semiconductor devices can be modulated, which is the basic principle of tribotronics.

Some literature focuses on the validation of the above tribo-potential modulation theory. Peng et al. establish a simulation model based on the finite element analysis. As is illustrated in Table 1, they find that when the distance between two tribo-layers is getting close to a certain value, *d*th, the voltage across gain and source of the TPG NMOS is approaching the threshold voltage *V*th of the FET, and the output curves of NMOS are very similar to the conventional MOSFET output characteristic [56]. Soon, the same phenomenon was discovered by Jiang’s team as well as by studying the performance of SG NMOS [57]. Additionally, works on logic devices such as the inverter [57,58,59,60] related to tribotronics also prove that tribo-potential can be used as an external gate voltage to control carrier transport in semiconductors, which can successfully replace the traditionally applied gate voltage.

**Table 1:** Comparison of experimental data and theoretical results illustrating the tribo-potential modulation effect.

Tribotronic Device	Parameter	Experimental Data	Theoretical Result
TPG NMOS [56]	—	*d* → *d*th Typical *I*_ds_-*V*_ds_ curves	*V*_gs_ → *V*th MOSFET output characteristic
SG NMOS [57]	—	*x* → *x*th Typical *I*_ds_-*V*_ds_ curves	*V*_gs_ → *V*th MOSFET output characteristic
Tribotronic inverter	*V*_OH_	4.933 V [58] 0.99 V [59] 1.0 V [60]	≥0.9 V [57]
	*V*_OL_	0.022 V [58] 0.01 V [59] 0.02 V [60]	≤0.1 V [57]

### 3.2. Tribotronic Field-Effect Transistor (TFET)

**Figure 2:**
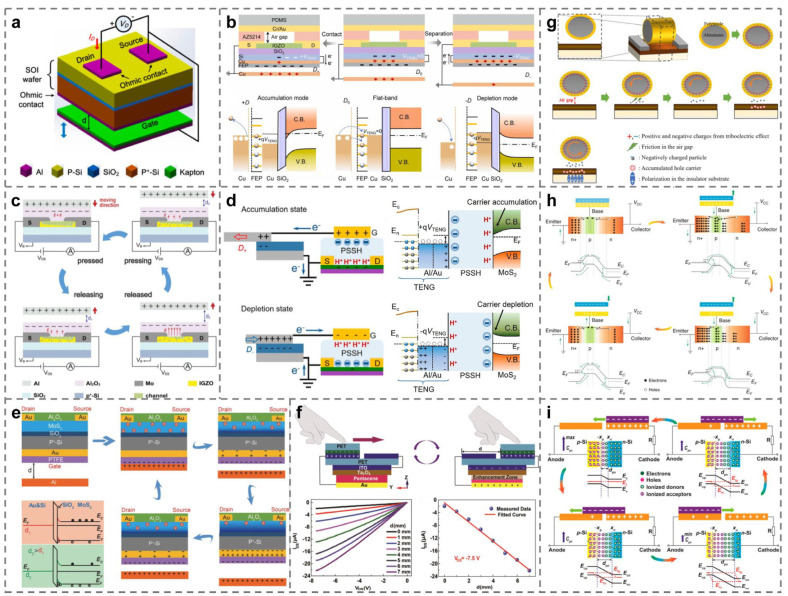
Typical tribo-potential modulated devices. (**a**) Contact electrification field-effect transistor (CE-FET) [14]. (**b**,**c**) Contact–separation mode tribotronic field-effect transistor (CS-TFET) [67,68]. (**d**) Lateral sliding mode tribotronic field-effect transistor (LS-TFET) [70]. (**e**) Single-electrode mode tribotronic field-effect transistor (SE-TFET) [73]. (**f**) Freestanding triboelectric-layer mode tribotronic field-effect transistor (FT-TFET) [75]. (**g**) Rotating gate tribotronic field-effect transistor (R-TFET) [88]. (**h**) Tribotronic bipolar junction transistor (TBJT) [89]. (**i**) Tribotronic tuning diode (TTD) [87].

In the field of microelectronics, metal–insulator–semiconductor (MIS) is a common capacitor structure, and it is also the basis of the metal-oxide–semiconductor field effect transistor (MOSFET) [61]. By using the tribo-potential generated by the contact between Kapton and Al foil to replace the original external gate bias, Zhang et al. successfully manufactured the first field effect transistor based on the contact electrification effect, namely CE-FET, as shown in Figure 2a. However, different from the “gate, drain, and source” three-terminal structure of traditional FET, CE-FET is only a two-terminal device. The external mechanical force that drives the contact–separation movement of the contact electrification layer replaces the third terminal of the traditional FET and directly generates a directional electric field inside the device to regulate the width of the conductive channel and the transportation characteristics of carriers therein. Table 2 shows the comparison between CE-FET and traditional FET. Compared with traditional FET devices, CE-FET can directly interact with the environment, and the tuning/control of its performance is not only related to the channel size, channel doping concentration, and gate capacitance of the FET itself but also affected by the maximum separation distance of the contact layers, the triboelectric charge density, etc. The switching speed of the CE-FET is slower than that of the traditional FET (only up to MHz) [14], but its material compatibility with FET devices, environmental interactivity, and other unique characteristics make it have a broad application prospect in human–machine interfaces, sensors, and other fields. This achievement is regarded as the beginning of the research on tribo-potential modulated devices, and then the concept of tribotronic field-effect transistors (TFET) is gradually introduced.

**Table 2:** Comparison of traditional FET and CE-FET [14].

Transistor Type	Traditional FET	CE-FET
Structure	3-terminal (Source, drain, and gate)	2-terminal (Source and drain)
Gate voltage	External voltage applied by the external circuit outside the FET	Contact voltage generated by the relative motion of the contact layers inside the CE-FET
Control	External circuit	Environmental, mechanical behavior
Performance controlled factor	Channel length, channel width, channel doping concentration, gate capacitance…	Vertical distance, triboelectric charge density, channel length, channel width, channel doping concentration, gate capacitance…
Switching speed	Fast (GHz)	Slow (MHz)
Applications	Amplification, variable resistor, electronic switch	Human/environmental interfacing, sensors, MEMS, flexible electronics, tribotronics, tribophotonics, tribo-phototronics, tribotromagnetism

In order to establish a more perfect and rigorous TFET theory, Liu et al. make a theoretical analysis of TFET for the first time by combining analytical calculation with numerical simulation. At the same time, the basic circuit of TFET is studied from two aspects of logic operation and mechanical sensing, which provides a complete theoretical basis and optimization strategy for designing such circuits [62]. Subsequently, Peng and his team used the method of finite element analysis (FEA) to study the influence of the relative distance between friction layers on the output current of a silicon-based N-channel MOSFET (NMOS) with the output of PTFE/Al CS-TENG as the gate control voltage. On this basis, using the coupling effect of the contact electrification effect and electrostatic induction effect, an NMOS with friction potential as both gate voltage and drain voltage is designed. This structure breaks away from the constraint that the original TFET still needs to apply drain bias voltage to generate current in the channel and theoretically realizes the full self-power supply of TFET for the first time [56]. The above achievements provide an in-depth understanding and design guidance for the potential applications of micro/nano electro-mechanical systems (MEMS/NEMS), human–computer interaction, flexible electronics devices, and self-powered active sensors. Inspired by this, in 2018, Jiang et al. also used finite element analysis (FEA) to study the enhanced and depleted MOSFET with the friction potential generated by the relative sliding of PTFE and Nylon as the gate control voltage. The simulation results show that the gate voltage of both P-channel and N-channel MOSFETs will increase with the increase in sliding distance between friction layers. Reflected in the final output, the channel current also increases gradually [57]. The above fruits further expand the basic theory of TFET, laying a foundation for developing TFET with more operating modes and corresponding circuit design.

With the deepening of research, the generation methods of TFET gate control voltage are gradually diversified. If the tribo-potential generated by TENG provides the gate control voltage, TFET can also be divided into the following four operating modes according to the different working modes of TENG: contact–separation mode tribotronic field-effect transistor (CS-TFET), lateral sliding mode tribotronic field-effect transistor (LS-TFET), single-electrode mode tribotronic field-effect transistor (SE-TFET), and freestanding triboelectric-layer mode tribotronic field-effect transistor (FT-TFET). The following will explain the principles of the above four TENG-gated tribotronic field-effect transistors, respectively.

#### 3.2.1. Contact–Separation Mode Tribotronic Field-Effect Transistor (CS-TFET)

CS-TFET is the most basic structure with the most research results [63,64,65,66]. Figure 2b,c shows CS-TFET driven by Cu/FEP CS-TENG and Al/Al_2_O_3_ CS-TENG as gate voltage sources, respectively [67,68]. The operating principle of CS-TFET can be explained by taking Figure 2c as an instance. In the initial state, the movable Al layer and Al_2_O_3_ layer are separated from each other, and the CE effect does not occur. Therefore, there is no friction charge on Al and Al_2_O_3_ layers at first and no modulation effect on channel current, either. When the two friction layers are in full contact with each other under the action of external mechanical force, the CE effect will occur. Negative charges will be formed on the surface of the Al_2_O_3_ layer with a stronger ability to attract electrons, while positive charges will be generated on the surface of Al foil. At this stage, because the distance between the two friction layers can be ignored, the negative charges on Al_2_O_3_ are balanced by the positive charges on Al foil, so the repulsion effect on carriers in the indium gallium zinc oxide (IGZO) semiconductor layer can be ignored, and the channel current has not changed significantly. Once the friction layers are gradually separated, the conservation of charge between the two friction layers will be broken, and some unrestricted negative charges on the Al_2_O_3_ layer will establish an internal electric field on the IGZO semiconductor layer, which will lead to the repulsive effect of carriers in the IGZO layer. Thus, during the release process, the carriers near the IGZO/Al_2_O_3_ interface decrease, which leads to the decrease in channel current. When the two friction layers approach each other again, as in the reverse process of the above-mentioned release process, the channel current will gradually increase until the two friction-charged layers contact again. Through the continuous circulations of the above process, the modulation effect of the contact–separation motion of the CS-TENG friction layer on the channel current of CS-TFET can be realized.

#### 3.2.2. Lateral Sliding Mode Tribotronic Field-Effect Transistor (LS-TFET)

Another widely studied is LS-TFET [59,69,70,71,72]. Cao et al. [68] use MoS_2_ as the conductive channel, PSSH as the gate dielectric, and output the tribo-potential through Al/PTFE LS-TENG as the gate voltage to control the movement of carriers in the FET channel, as shown in Figure 2d. The working mechanism of this structure can be described as follows: When Al foil and PTFE film are in contact with each other and in a state of gradual separation (*D*_+_), the positive friction charges induced in Al foil will be transferred to the gate of FET without limitation, resulting in a positive gate voltage. The transferred positive charges repel protons to the PSSH/MoS_2_ interface and induce the electron density in the channel to increase gradually, i.e., the accumulated state. However, when the state (*D*_−_) in which Al foil and PTFE film are close to each other is applied to TENG, more positive friction charges will be confined to the Al/PTFE interface, resulting in more reverse electrons being transferred to the gate of FET, which is equivalent to applying a negative gate voltage. The transferred electrons attract most protons to the Au/PSSH interface, thus reducing the electron density in the channel, that is, the depleted state. Based on the above two channel states, if an appropriate voltage bias is further applied to the drain of the FET, the sliding motion of the LS-TENG friction layers can control the performance of the LS-TFET.

#### 3.2.3. Single-Electrode Mode Tribotronic Field-Effect Transistor (SE-TFET)

In addition to the mentioned above two types of TFET, SE-TFET attracts researchers’ interest as well [73,74]. Figure 2e shows a MoS_2_-channel FET driven by Al/PTFE SE-TENG as the gate. In the initial state, PTFE and Al are in complete contact with each other, and charge transfer occurs. According to the triboelectric sequence, electrons will be injected into PTFE from Al, leaving positive triboelectric charges on the Al surface and negative triboelectric charges on PTFE. At this time, the friction charges with opposite polarities are completely balanced, and there is no influence on the width of the conductive channel. Once the positively charged Al is vertically separated from PTFE by a certain distance, some negative friction charges on the surface of the PTFE film will be out of restraint, and an internal electric field will be generated between the grounded source electrode and the gate electrode, which is equivalent to applying a negative voltage on the gate of a traditional MoS_2_ transistor. Therefore, some free electrons are depleted in the MoS_2_ channel, reducing the electron density and the corresponding drain-source current. After reaching the maximum separation distance, Al moves in the opposite direction and gradually approaches PTFE. The induced negative gate voltage decreases, which leads to an increase in channel width and the drain-source current. When Al and PTFE film contact again, the negative charges on the PTFE surface are completely balanced by the positive charges on the Al surface again, which no longer controls the MoS_2_ channel, so the carrier density and drain-source current return to the initial state. The above is the basic operating principle of SE-TFET.

#### 3.2.4. Freestanding Triboelectric-Layer Mode Tribotronic Field-Effect Transistor (FT-TFET)

Due to the high manufacturing difficulty and few application scenarios, there are few reports on the research results of FT-TFET [75,76]. Taking the FT-TFET shown in Figure 2f as an example, in the initial position, the FEP film is in close contact with the left ITO electrode. At this time, electrons are injected into the surface of the FEP film from the ITO electrode on the left, leaving positive charges on the ITO electrode and negative charges on the FEP film. The positive and negative charges on the surfaces of the two friction layers are completely balanced. The gate voltage is zero, and no conductive channel is formed in the FET. When the FEP film is driven by an external force to slide towards the right ITO electrode, the negative charges on the FEP film will induce electrons to flow from the right ITO electrode to the ITO gate electrode. At the same time, the positive charges on the left ITO electrode induce electrons to flow from the source electrode to the left ITO electrode. Therefore, a gate voltage is generated between the gate and the drain of TFET, and a conductive channel is formed, resulting in a drain-source current. However, when the FEP film slides back to its original position under the action of an external force, the balance of friction charges makes electrons flow back from the ITO gate electrode to the right ITO electrode, resulting in the gate voltage changing back to zero. This will reduce the width of the conductive channel and the drain-source current to the initial state. Therefore, the tribo-potential generated by FT-TENG can modulate the drain-source current of FT-TFET like the traditional gate voltage.

#### 3.2.5. Typical Performance Indicators of TFETs

Typical performance indicators of the four-mode TFETs above are also worth being noticed, and the relevant comparison can be found in Table 3. As is shown, the operating current of CS-TFET usually has a wider range than the other three modes, from 2 μA to 10 mA, while that of LS-TFET is restricted to only 0.5~20 μA. Meanwhile, CS-TFET can reach the maximum voltage of 10 V when working normally, which is also the highest among the four modes. Constrained by the architecture, the operating voltage of LS-TFET and SE-TFET is just around 1 V. When used as a smart sensor, the response time of the four TFETs is relatively close to each other, with the order of magnitude at hundreds of milliseconds. As for durability, due to no relative sliding process and low wear, the CS-TFET is able to operate regularly for nearly 10,000 cycles and still provide a satisfactory accuracy, which performs much better than the LS-TFET, SE-TFET, and FT-TFET.

### 3.3. Other Tribotronic Devices

Inspired by the above traditional TFET, some more interesting works on tribotronic devices have been reported in recent years. For example, team Shin uses the Al roller coated with polyimide (PI) as the gate, proposing an air-friction-driven rotating gate translator (AFRGT) [88]. As shown in Figure 2g, when the external force drives the roller to rotate around the central axis, friction occurs between PI and air, negatively charged particles are generated in the air gap, and carriers are induced in the adjacent active layers. With the increase in the rotating speed of the roller, a conductive channel rich in carriers is formed on the surface of the substrate. Under the action of external drain bias, drain-source current will be generated in the above channel. Xi et al. combined bipolar junction transistors (BJTs) with NPN structures with TENG theory and skillfully used the tribo-potential generated by Cu/FEP CS/LS-TENG as the base voltage of BJT to control the bias of the emitter junction and collector junction, as shown in Figure 2h. This achievement realizes the direct control of the output state of BJT by TENG and introduces the concept of tribotronic bipolar junction translator (TBJT) [89]. Similarly, as shown in Figure 2i, Zhou and his team members invent a tribotronic tuning diode (TTD). The forward bias/reverse bias of the diode is controlled by TENG’s output tribo-potential, and an adjustable capacitor controlled by external force is realized, which has been successfully applied to the analog signal filtering circuit [87].

In addition, the voltage generated by the tribovoltaic effect also modulates the carrier motion in semiconductors, which brings new inspiration to the study of tribotronics devices. For example, Luo et al. constructed a tribovoltaic nanogenerator using an MXene layer and Si wafer (named MS-TVNG) and proposed MS-TVNG-based sensors for velocity, displacement, tension, oscillation angle, and vibration, which provide great opportunities for research and engineering applications of new devices based on the principle of the tribovoltaic effect [90]. Huang et al. proposed a flexible liquid-based continuous direct-current tribovoltaic generator (FLG), which can be used as a multifunctional self-powered sensor to detect temperature, pressure, and positional attitude, providing an effective method for designing flexible continuous DC generators as well as new multimodal sensors based on liquid DC generator mechanisms [35]. However, most of the current research on tribovoltaic focuses on exploring the mechanism [33,34,91], optimizing the structure [92,93,94], and improving the output performance [95,96,97,98], etc., while less research has been conducted on its application in tribotronic devices. If the tribovoltaic effect of semiconductor materials can be effectively combined with tribotronics in the future, it is foreseeable that this can reduce the complexity of tribotronics devices to a certain extent and enrich their application scenarios.

## 4. Tribotronic Applications

Tribotronic devices have a very broad application prospect because of their wide range of available materials, diverse operating modes, modular customization, high compatibility with semiconductor processes, and easy large-scale integration. At present, tribotronic devices have been widely studied in logic circuits and memory devices, smart sensors, artificial synapses in human–computer interaction systems, etc. Quite a few inspiring achievements have been made, and the application modes of tribotronic devices are gradually diversified and refined.

### 4.1. Logic Circuits and Memory Devices

**Figure 3:**
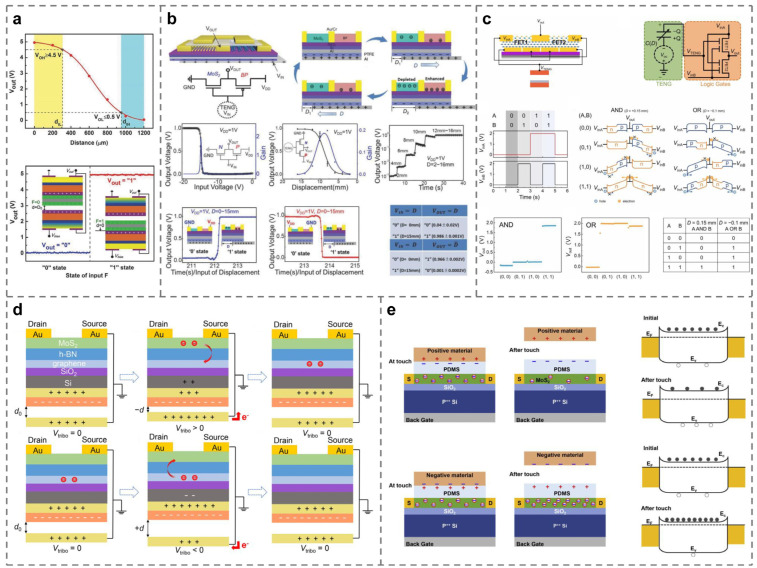
Tribotronic logic circuits and memory devices. (**a**–**c**) Logic circuits composed of TFETs produce different logic outputs [58,59,60]. (**d**,**e**) TFETs equipped with floating gates to realize the basic function of memory devices [101,102].

The logic circuit is the first field where tribotronic devices are put into practical application. Figure 3a–c shows several typical logic circuits made of TFETs [58,59,60]. Shortly after putting forward the concepts of CE-FET and tribotronics, the Zhang crew applies CS-TFET to the logic circuit for the first time and successfully outputs the expected logic signal [58]. As shown in Figure 3a, two identical floating contact-electric-field gated tribotronic transistors (CGT) are connected in reverse, and a double-layer Al/PTFE CS-TENG is shared as the gate voltage source of the two CGTs. When the PTFE layer is in contact with the upper or lower Al gate during the movement, the upper and lower CGTs can be controlled to be turned on or off, thus generating a logic level output of “0” or “1” in the external circuit and realizing the NOT gate in the traditional CMOS circuit. Subsequently, based on this basic structure, by adding more CGTs for proper circuit connection, basic logic functional gates such as AND, OR, NAND, NOR, XOR, and XNOR are also developed. In 2017, Zhang et al. integrated the above discrete logic gates with traditional digital circuits and manufactured TFET-based S-R flip-flops, D flip-flops, and T flip-flops. In addition, simple tribotronic sequential logic circuits, such as registers and counters, are integrated to realize the storage and calculation triggered by external contact [99]. Gao and his colleagues are committed to manufacturing double-gate TFET to improve the on/off ratio and reduce the off current. That is a tunable triboelectric dual-gate logic device based on N-type MoS_2_ FET, P-type black phosphorus (BP) FET, and LS-TENG, as shown in Figure 3b. The electrical properties of this double-gate TFET are characterized in detail, and the figure-of-merits of triboelectric logic devices are put forward for the first time, including triboelectric transconductance, triboelectric subthreshold swing, and triboelectric logic gate power consumption [59]. In recent years, some researchers have integrated two TENG’s into two discrete gates of MoTe_2_ FET, which further improves the integration of digital logic circuits based on TFET and reduces the overall power consumption [60], as shown in Figure 3c.

If the floating gate is added to the basic TFET structure, a tribotronic memory device, as shown in Figure 3d,e, can also be manufactured. By controlling the generation/disappearance of the tribo-potential through mechanical signals, the memory can be programmed/erased [100], and even in the programming stage, completely zero power consumption can be realized [101]. Jia et al. reported a new type of multi-bit tribotronic nonvolatile memory (T-NVM), which has a large memory window of 60 V, an on/off current ratio as high as 105, and a retention time of over 6000 s [102]. In 2021, Zhao’s team couples tribotronics with photoelectric effects to prepare a multi-bit nonvolatile tribo-photoelectric double-gate memory. In the programming process, TENG’s mechanical motion and incident light can both adjust the storage state by tunneling electrons into the MoS_2_ channel. On the contrary, the reverse movement of TENG can tunnel electrons back to the charge-trapping layer, thereupon completely erasing the data [103]. The above research further expands the application scenarios of tribotronic memory devices.

### 4.2. Smart Sensors

**Figure 4:**
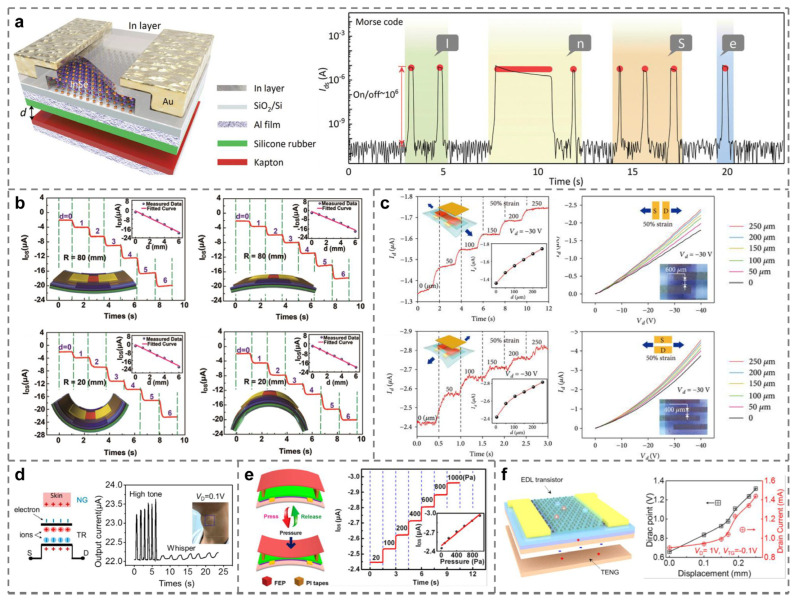
Tribotronic Smart Sensors. (**a**) Through tactile sensing, the Morse code of “InSe” is demonstrated [78]. (**b**,**c**) Relationship between the drain-source current and the bending or strain deformation detected by tribotronic strain sensors [75,83]. (**d**) Sensations of the tone with a tribotronic voice sensor [82]. (**e**) Structure and performance of a flexible organic tribotronic transistor (FOTT) for pressure sensing [77]. (**f**) An electric double layer (EDL) TFET for accurate displacement sensing [85].

Smart sensing is the closest to daily life and the most fruitful research direction in the field of tribotronics [68,72,74]. The first application of tribotronic devices in smart sensing can be traced back to 2015. It is a tactile sensor made by Xue et al. by using a single SE-TFET [73]. Subsequently, based on the above tactile sensing technology, Yang et al. use several TFETs driven by PTFE/Cu CS-TENG for large-scale integration, further optimizing the sensitivity and accuracy of the tactile sensor [79]. With the development of the research process, Li et al. innovatively realized the ideal logical operation through tactile perception and simulated the Morse codes “I”, “n”, “S”, and “e” of the two main elements In and Se, as shown in Figure 4a [78]. However, the above literature on tactile sensing is mostly limited to the sensing of vertical pressure, which limits the potential application prospects. Therefore, researchers gradually focus on using tribotronic devices to sense strains in more directions [84]. In Figure 4b, Pang and his team explore the output current of flexible TFET composed of organic thin film transistors (OTFTs) and FT-TENG under different bending modes and degrees. They find that with the gradual increase in bending radius, the channel current of the device also increases regularly [75]. Similarly, in 2020, team Zhao proposed a stretchable organic tribotronic transistor (SOTT), which can be stretched in parallel and perpendicular to the channel direction. Under the tensile action in the above two directions, the device has excellent performance in the strain range of 0 to 50%, as shown in Figure 4c [83]. Finally, tribotronic devices have been developed for other interesting sensing purposes, such as judging the tone of the volunteer when speaking by driving the flexible CS-TFET through the movement of the larynx [Figure 4d] [82], corresponding the applied pressure with the drain-source current of TFET, and making regular numerical analysis [Figure 4e] [77], or accurately sensing the relative displacement of the object [Figure 4f] [85]. Introducing tribotronics into the field of smart sensing not only greatly improves the sensing efficiency but also further reduces energy consumption and opens up a new research direction called “self-powered sensing”.

For different purposes, tribotronic transistors have different materials, structures, and properties. In terms of environmental sensors, a number of researchers have developed sensors for temperature, pressure, illumination, magnetic field, and other environmental conditions, respectively. Comparisons of the performance of tribotronic transistors in different environmental scenarios are shown in Table 4.

Among them, Meng et al. developed graphene-based TFET (named GSFET and GFET, respectively). Although they are both CS-TFET, GSFET shows a larger change in I_D_ than GFET in the temperature range of 25~45 °C, i.e., GSFET is more sensitive to temperature, demonstrating the potential of the GSFET active matrices for self-powered E-skin applications [81]. In terms of pressure sensors, Tan et al. integrated a sensitive pressure sensor based on the capacitance change in an air-dielectric top gate upon external pressure, which can respond to small pressures ranging from 55 to 344.4 kPa with a short response time (≈120 ms) and offers an effective solution to tunable or multifunctional pressure/tactile sensors [67]. Bu et al. realized the nanoscale triboelectric modulation on electronics using contact-mode atomic force microscopy (AFM) and scanning Kevin probe microscopy (SKPM), where I_D_ increases upon contact force increase from 0 to 2.5 nN and saturates at contact forces greater than 2 nN, which provide a deep understanding for the theoretical mechanism of tribotronics and may have great applications in a nanoscale transistor, micro/nano-electronic circuit, and NEMS [86]. The flexible organic tribotronic transistor (FOTT) developed by Zhao et al. is excellent in pressure sensing with high sensitivity (maximum sensitivity of 21% Pa^−1^), a fast response time (≈110 ms), and excellent stability, demonstrating bright potentials of tribotronics in human–machine interactions, smart skins, wearable devices, intelligent sensing, and so on [77]. In the studies about photodetectors, Zhang et al. demonstrated triboelectric-charge-enhanced photoelectric conversion using TPT, and the photocurrent has a linear relationship with the illumination and is increased with the decreasing distance of CS [104]. Pang et al. dramatically increased the photoresponsivity of the MoS_2_ phototransistor to 727.8 A/W at the 100 mW/cm^2^ UV excitation intensity and 1 V bias voltage by coupling 2D material-based photonics with tribotronics [69]. In addition, the GFET by Meng et al. used a magnetic film and an ion gel as the friction layers, and when the magnetic field intensity was increased from 7 mT to 14.6 mT at a given *V*_D_ of 0.1 V, the magnetic field stimulation changed the distance between the magnetic film and the ion gel, resulting in a rate of change in *I*_D_ of 0.66 µA/mT, which suggests that it can be used to simulate the skin’s reactivity to magnetic stimulation [80]. When used for magnetic sensing, the FOTT developed by Zhao has a sensitivity of 16% mT^−1^, a response time of 120 ms, and excellent stability of more than 10,000 cycles when the sensor is operated at 1~150 mT, which bodes well for the bright future of such magnetic sensors for smart sensing and control [77].

Few studies have mentioned the application of tribotronic devices in humidity detection. If considered the combination of liquid–solid mode TENG with FET devices or the use of moisture-sensitive materials (such as MoS_2_, graphene and its derivatives, carbon nanotube, ZnO, etc.) as the friction layer materials, it may be expected to make a new breakthrough in electronic skin, weather monitoring, intelligent recognition, etc.

**Table 4:** Comparison of the performance of tribotronic transistors in different environmental scenarios.

Application	TFET Type	Test Condition	Range of *I*_D_ (Δ*I*_D_)	Setting Condition
Temperature sensor	CS-TFET [81] (GSFET)	25~45 °C	252~277 μA (25 μA)	*V*_D_ = 0.5 V
	CS-TFET [81] (GFET)	25~45 °C	78.5~82.4 μA (3.9 μA)	*V*_D_ = 0.5 V
Pressure sensor	CS-TFET [67] (Triboelectric potential tuned dual-gate IGZO transistor)	55.5~344.4 kPa	1.9~3.2 nA (1.3 nA)	*V*_D_ = 15 V *D*_CS_ = 0.07 mm
	LS-TFET [86] (NTT)	0~2.5 nN	139~272 μA (133 μA)	—
	CS-TFET [77] (FOTT)	20~1000 Pa	≈ 0.52 μA	*h*_FEP film_ = 100 μm
Photodetector	CS-TFET [104] (TPT)	0~1 mW	0~16 μA (16 μA)	*λ* = 680 nm *D*_CS_ = 2 mm
	LS-TFET [69] (MoS_2_ phototransistor)	10~400 mW/cm^2^	2~4 μA (2 μA)	*V*_D_ = 1 V *D*_LS_ = 8 mm
Magnetic field sensor	CS-TFET [80] (GFET)	7~14.6 mT	25.1~20.1 μA (5 μA)	—
	CS-TFET [77] (FOTT)	1~150 mT	−2.44~−2.83 μA	*V*_D_ = 0.1 V *h*_PDMS/Fe3O4 film_ = 250 μm

### 4.3. Artificial Synapses

**Figure 5:**
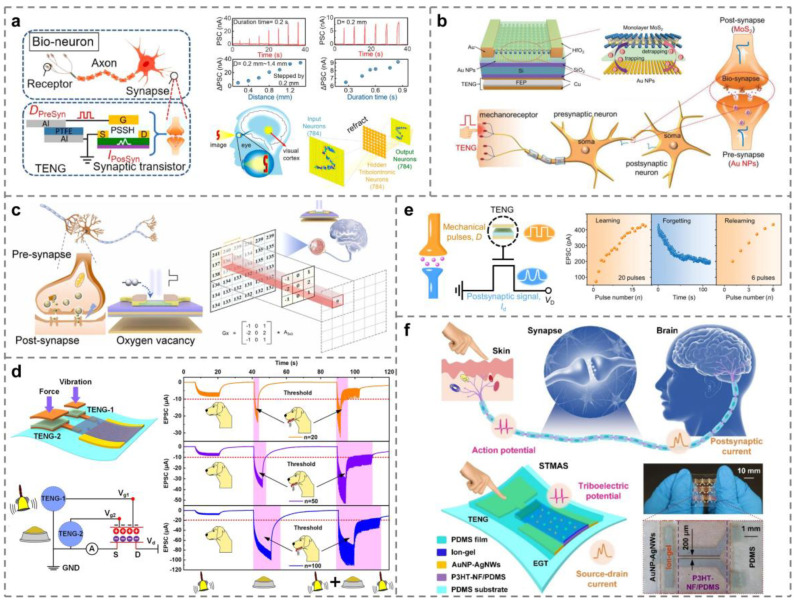
Tribotronic artificial synapses. (**a**) A visual imaging system formed by artificial synapses based on the TFET [70]. (**b**) An artificial synapse unit composed of mechanoplastic floating-gated neuromorphic MoS_2_ TFET in comparison with the biological nerve system [105]. (**c**) The artificial photonic synaptic based on IGZO TFET to perform image edge detection [67]. (**d**) Artificial synapse-like simulation of associative memory in Pavlov’s dog [106]. (**e**) Emulation of the learning-experience behavior of a plastic artificial synapse [107]. (**f**) Tribotronic artificial synapses used for a human tactile sensory nervous system [108].

With the acceleration of industrial automation, artificial intelligence technology is constantly developing and innovating, and research in the field of human–machine interaction has gradually become a hot spot. The artificial synapse used to replace the human brain for calculation and reasoning is the key research direction in the future. Around 2020, Yang et al. first applied tribotronic devices to artificial synapse simulation. As shown in Figure 5a, TENG can simulate mechanical receptors to transmit presynaptic signals, while PSSH-gated TFET can work as a postsynaptic component and realize corresponding commands through postsynaptic current (PSC), applied to the visual imaging system [70]. Subsequently, the team further optimizes the above result and proposes a versatile mechanical artificial synapse. An artificial neural network can be constructed by using three parallel artificial synapses, which can realize the mechanical plasticity of a neuromorphic logic switch, overcome the von Neumann bottleneck, and perform neuromorphic logic conversion and data storage at the same time [Figure 5b] [105]. Based on the basic theory of a tribotronic artificial neural network, Tan et al. realize the image edge detection system based on an artificial photonic synapse, as shown in Figure 5c [67]. By 2023, Zeng’s results on artificial synapses are really refreshing. As shown in Figure 5d, researchers use vibration-driven TENG-1 as the input source of “bell” and force-driven TENG-2 as the input source of “food”, combined with TFET to simulate Pavlov’s dog experiment. The results show that tribotronic artificial synapse (TAS) can effectively imitate conditioned reflex [106]. Similarly, Jia and others successfully simulate the experiment of learning-experience behavior [Figure 5e] [107]. Zeng et al. also realize the human tactile sensory nervous system using stretchable tribotronic mechanical artificial synapses (STMASs) [Figure 5f] [108]. A variety of neural systems based on artificial synapses can be organically combined with each other to form a huge neural network for simulating the calculation of the human brain, which has created a new era of artificial intelligence research.

### 4.4. Applications Coupled with Other Effects

**Figure 6:**
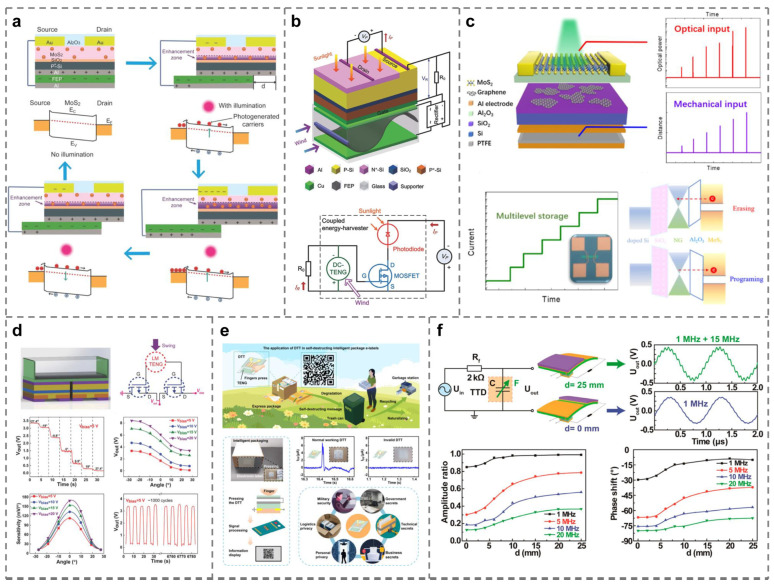
Tribotronic applications are coupled with other effects. (**a**) A photoresponsivity-enhanced MoS_2_ tribotronic phototransistor [69]. (**b**) The hybrid energy harvesting system consists of a TFET and a photodiode for simultaneously scavenging wind and solar energies [104]. (**c**) A multibit nonvolatile optoelectronic memory that can store both optical and mechanical stimulation information [103]. (**d**) The liquid metal-gated TFET is suitable for an electronic gradienter [109]. (**e**) TFETs used for degradable intelligent package e-labels [110]. (**f**) The tribotronic tuning diode (TDD) serving as a signal modulation component [87].

In some cases, researchers will also couple tribotronics with other effects in order to obtain more outputs that meet specific conditions. For instance, three different groups of researchers have the inspiration of combining tribotronics with photoelectronics and obtain the following results, respectively: First, a MoS_2_ tribotronic phototransistor is proposed, which can greatly enhance the light response characteristics, as shown in Figure 6a [69]. Second, the output voltage of TENG driven by the wind on the external resistor is used as the gate voltage of TFET to improve the conversion efficiency from solar energy to electric energy during the operation of the field-effect phototransistor, as shown in Figure 6b [104]. Third, a tribo-phototronic nonvolatile memory adjusted by incident laser intensity is put forward, as shown in Figure 6c [103]. In addition, in 2018, Bu et al. prepared another electronic gradienter, as shown in Figure 6d, for angle measurement, linking the CE effect of liquid–solid with TFET [109]. Finally, there are also tribotronics and tribotronic devices in the innovative fields, such as intelligent e-labels [Figure 6e] [110] and signal modulation [Figure 6f] [87]. The combination of tribotronics and related fields is increasingly close to the development of the research process.

## 5. Summary and Perspectives

Since the basic concept was put forward in 2014, tribotronics and related fields have been widely concerned and studied. Meanwhile, the basic knowledge system of tribotronics has been gradually established. This paper systematically summarizes the research progress of tribotronics at first. Based on the contact electrification (CE) effect and the theory of triboelectric nanogenerator (TENG), tribotronics controls the carrier transportation characteristics in semiconductors through the tribo-potential introduced by TENG, thus regulating the output characteristics of semiconductor devices and realizing various practical functions. Then, grounded by the contact electric field effect transistor (CE-FET), the tribotronic field-effect transistor (TFET) with tribo-potential as the gate voltage is introduced. According to the different working modes of TENG, four operating principles of TFET are classified and described. Finally, the potential applications of tribotronics in logic circuits and memory devices, smart sensors, and artificial synapses are reviewed.

Although considerable research progress has been made, as an emerging field, tribotronics still faces many difficulties and challenges in order to adapt to the developing contemporary society and meet the increasingly diversified needs in production and life. In the future research process, in order to realize higher performance and more practical tribotronic devices, several detailed suggestions will be put forward here for the development of tribotronics from the following aspects.

Device simulation. At present, the simulation fruits of tribotronics are still very limited. The relatively simple practical operation experiment and test generally have the problem of high material and time cost, which further restricts the future applications of tribotronic devices. However, some problems can be avoided by early device simulation, which provides top-level guidance for the actual preparation of devices and improves efficiency. It is suggested to explore the possibility of practical application of tribotronic devices under more physical environment conditions by using multi-physical field simulation tools such as COMSOL.

Device performance optimization. Improving the performance of tribotronic devices, especially the durability and stability, is a significant issue that researchers need to well consider. Considering that tribotronic devices are mostly composed of TENG and FET, the performance of devices can be optimized from two aspects, as follows: On the one hand, materials that are far away from each other in the triboelectric sequence can be properly selected as TENG friction layers, resulting in a larger triboelectric potential and expanding the dynamic range of normal operation. At the same time, materials should be developed or used with better wear resistance to prolong the service life of devices. On the other hand, the semiconductors with higher carrier mobility should be selected as the conductive channel of FET, which strengthens the regulation of tribo-potential on carrier transportation state and further improves the overall controllability. In addition, the aging process in the microelectronics industry can be applied as a reference to upgrade the stability of the device.

Array integration. At present, TFET, TBJT, TTD, and other tribo-potential modulated devices operate discretely due to their relatively larger size compared with traditional semiconductor devices, resulting in the defects of single function and difficult integration. The sensitivity and accuracy are also too limited to meet the actual demands in industries and lives when only a single device is applied. Therefore, it is essential to further miniaturize the existing tribotronic devices to satisfy the needs of large-scale array integration and system synthesis in the future so as to overcome the shortcomings, improve the performance, and gradually solve the above problems.

Scalability enhancement. In most current application scenarios, multi-functional products are urgently required to establish a robust system, which calls for higher scalability of the tribotronic devices. An effective method is trying to couple tribotronics with other semiconductor-related effects, such as tribovoltaic effect, piezoelectric effect, and pyroelectric effect, to design more novel and multipurpose devices. Another concern that should be paid great attention to is the compatibility of different tribotronic components during the scalability process. Appropriate device arrangement and good impedance matching will not only help increase the energy transmission efficiency of the whole system but decrease the power dissipation as well.

Cost reduction. In traditional technology, in order to realize the multi-function of devices, it is usually necessary to manufacture a series of single-functional devices first and then integrate them into one or several systems. The production cycle and product cost in the above process are generally high, which restricts the high integration of devices and is not conducive to large-scale industrial production. Thus, how to reduce the cost is an important problem to be solved in the process of putting tribotronic devices into practical application as soon as possible. At present, exploring and utilizing fourth-generation semiconductors to replace conventional ones, such as AlN, Ga_2_O_3_, and diamond, is a promising orientation worthy of consideration in the future. At the same time, optimizing the device structure, improving the production process, and reinforcing the level of automation can also be used to further reduce the production cost.

In conclusion, tribotronics is inspired by the CE effect, and through TENG and FET as intermediaries, the tribo-potential is truly combined with practical application in an ingenious way. The potential application of micro/nano energy in logic storage, self-powered sensing, human-machine interaction, flexible electronics, and intelligent manufacturing is further explored, which brings a bright prospect for intelligent life and industrial automation.

## Figures and Tables

**Table 3:** Comparison of key performance indicators of different mode TFETs.

Performance Indicator	CS-TFET [67,68,77,78,79,80,81,82,83,84,85]	LS-TFET [59,69,70,71,72,86]	SE-TFET [73,74]	FT-TFET [75,76,87]
Operational current	2 μA~10 mA	0.5~20 μA	15~300 μA	20~30 μA
Operational voltage	0.1~10 V	0.1~1.5 V	0.5~1 V	1~8 V
Response time	10~150 ms	150~800 ms	30~400 ms	300~500 ms
Durability	1000~10,000 cycles	20~100 cycles	1000~2000 cycles	1000 cycles
